# Unraveling the biological functions of Smad7 with mouse models

**DOI:** 10.1186/2045-3701-1-44

**Published:** 2011-12-28

**Authors:** Lu Zhu, Susie Chen, Yan Chen

**Affiliations:** 1 Key Laboratory of Nutrition and Metabolism, Institute for Nutritional Sciences, Shanghai Institutes for Biological Sciences, Graduate School of the Chinese Academy of Sciences, Chinese Academy of Sciences, Shanghai, China

## Abstract

Smad7 is a key negative regulator of the transforming growth factor β (TGF-β) signaling and plays an important role in modulating a large array of biological processes. The physiological actions of Smad7 have been extensively investigated by using various mouse models. These studies have pinpointed numerous important *in vivo *functions of Smad7, including its activity in early embryonic development, fibrosis of many organs, skin cell differentiation, regulation of immune response and inflammation, tumorigenesis, and metabolic control. As most biological activities modulated by Smad7 are closely related to human disorders, it is anticipated that Smad7 will continue to be an intriguing molecule that will be vigorously investigated in the future to strengthen our understanding about the pathogenesis of human diseases.

## Introduction

Members of the transforming growth factor β (TGF-β) superfamily play essential roles in many physiological and biological processes including embryonic development, tumorigenesis, immunity, inflammation, and various physiologic courses [1]. The canonical signaling event induced by TGF-β superfamily members is initiated by ligand-mediated heteroligomerization of the serine-threonine kinase receptors, which phosphorylate signaling-restricted Smads (R-Smads), such as Smad2/3 for TGF-βs and Smad1/5/8 for bone morphogenetic proteins (BMPs). The activated R-Smads then form a heterocomplex with a common Smad (Co-Smad or Smad4) and the complex is translocated into the nucleus, resulting in transcriptional regulation of a broad repertoire of target genes [2,3]. On the other hand, Smad6 and Smad7 fall into the inhibitory Smad (I-Smad) subfamily that contains Smad6, a relatively specific inhibitor for BMP signaling, and Smad7, a general inhibitor for both TGF-β/activin signaling and BMP signaling [4-6]. Originally, Smad6 and Smad7 were discovered to be upregulated by shear stress in human vascular epithelium [7]. Smad7 was first reported as an inducible antagonist for TGF-β-mediated signaling upon TGF-β activation [5]. It was found to stably associate with the TGF-β receptor complex and block phosphorylation of Smad2/3 upon TGF-β stimulation [5]. Smad7 antagonizes TGF-β signaling through multiple mechanisms, such as by interfering with the recruitment of R-Smads upon TGF-β/activin activation [5,8], by inducing degradation of TGF-β type I receptor after recruitment of E3 ubiquitin ligases [9-11], and by recruiting protein phosphatase 1 (PP1) to the receptor complex via interaction with growth arrest and DNA damage protein 34 (GADD34) [12]. In addition to its inhibitory activity to TGF-β/activin signaling, Smad7 was also found to be actively involved in mediating the cross-talk between TGF-β signaling with other cellular signaling pathways [13].

Since the identification of Smad7 fourteen years ago, the physiological functions of Smad7 have been uncovered mainly through two lines of studies. Firstly, dysregulation of Smad7 has been demonstrated to be associated with the pathogenesis of many human diseases, including malignancy, scleroderma, and chronic inflammatory bowel disease (IBD). Secondly, various animal models have been used to unravel the physiological activities of Smad7. In this review, we will mainly focus on those discoveries pertinent to the physiological functions of Smad7 by studies using mouse models.

### Smad7 and embryonic development

The effect of Smad7 on mouse development has been revealed by manipulation of Smad7 expression through transgenic overexpression, virus-mediated transfection and genetic deletion by knockout technology. Overexpression of Smad7 in mouse zygotes inhibits embryonic development beyond the 2-cell stage [14]. Adenovirus-induced overexpression of Smad7 into embryonic mouse lungs in culture prevents TGF-β-mediated inhibition of both lung branching morphogenesis and epithelial cell differentiation, indicative of potential activity of Smad7 in lung development [15]. Transgenic overexpression of Smad7 in the mouse skin disrupts hair follicle morphogenesis and induces premature sebaceous gland development by antagonizing Wnt/β-catenin signaling [16]. It was also reported that Smad7 inhibits chondrocyte differentiation possibly by down-regulating BMP-activated p38 mitogen-activated protein kinase (MAPK) pathways [17]. Furthermore, neural crest-restricted Smad7 overexpression leads to defects in craniofacial and cardiovascular development [18]. Collectively, while these overexpression-based studies may indicate that Smad7 has a function in early development, none of these studies could provide a definitive answer about the *bona fide *activity of Smad7 during early development.

Currently, there are two reported mouse models with embryonic deletion of Smad7 [19,20]. A hypomorphic Smad7 mouse model was generated by Li *et al. *[19], in which the exon 1 region of the Smad7 gene was deleted. These mutant mice appeared to have normal phenotypes, except for minor alterations in B-cell responses [19], likely due to the observation that the Smad7 protein deleted of the Mad-homology 1 (MH1) domain encoded by exon 1 retains the inhibitory activity on TGF-β signaling [21]. The second Smad7 knockout model was generated by depletion of exon 4 that encodes MH2 domain of Smad7 protein and such deletion completely abolished the inhibitory activity of Smad7 on TGF-β signaling [20]. The majority of the Smad7 mutant mice with exon 4 deletion died in *utero *with multiple developmental defects in the heart, including ventricular septal defect (VSD) and noncompaction of myocardium [20]. Only a small percentage of these Smad7-deficient mice could survive to adulthood, accompanied by a severe decline of cardiac functions and arrhythmia [20]. Therefore, it appears that the major effect of Smad7 on embryonic development is restricted to the cardiovascular system, especially the tissues originated from the endocardial cushion, as Smad7 was found to be highly expressed in the cushion region [22].

### Smad7 and fibrosis

TGF-β has long been considered to be a master mediator of fibrosis in many organs including the lung, kidney, pancreas, eyes, and liver. The established understanding of fibrogenesis by TGF-β signaling is initiated by the activation of Smad-mediated pathway upon ligand binding, followed by myofibroblast transformation and stimulation of the synthesis of extracellular matrix (ECM) proteins [23]. Various fibrogenic factors have been demonstrated to participate in these processes, including type I collagen, fibronectin, laminin, and matrix metalloproteinases (MMPs). As the critical negative modulator of TGF-β signaling, Smad7 has been proposed to play a pivotal role in the protection against TGF-β-induced fibrosis [24,25].

In the mice, overexpression Smad7 was first reported to prevent bleomycin-induced lung fibrosis in an adenovirus-mediated gene transfer model [26], suggesting that Smad7 may have applicability in the treatment of pulmonary fibrosis. During the development of unilateral ureteral obstruction (UUO) of kidneys in mice, a model of progressive tubulointerstitial fibrosis, the protein level of Smad7 was found to be progressively reduced [27]. Interestingly, the reduction of Smad7 in UUO kidneys was found to be caused by an increase in E3 ubiquitin ligase and accelerated degradation of Smad7 protein [27]. Consistently, deletion of Smad7 in the mice was found to promote renal fibrosis in UUO kidneys [28]. In addition to its effect on renal fibrosis, Smad7 has been shown to play a protective role in fibrosis in other organs. Pancreatic fibrosis is the hallmark of chronic pancreatitis, currently an incurable disease. Smad7 also possesses a protective effect on cerulein-induced pancreatic fibrosis as revealed by overexpression of Smad7 in the pancreas using an elastase promoter [29]. Furthermore, it was found that Smad7 overexpression could protect retinal pigment fibrosis in a proliferative vitreoretinopathy mouse model [30].

Fibrosis has also long been considered a leading cause of liver diseases following chronic liver injury. The final common pathway of liver fibrosis is cirrhosis, characterized by accumulation of fibrillar interstitial collagens type I and III, liver failure, and portal hypertension. Dysregulated TGF-β signaling is implicated in chronic liver diseases [31]. In the mice, either overexpression or down-regulation of Smad7 in the liver has been used to investigate the effect of Smad7 on liver fibrosis. Ablation of TGF-β signaling by overexpression of Smad7 specifically in hepatocytes is sufficient to blunt the fibrogenic response after tetrachloride (CCl_4_) challenge and attenuate liver damage [32]. On the other hand, reduction of Smad7 expression could accelerate CCl_4_-induced liver damage and fibrogenesis [33]. Collectively, these studies have corroborated the protective function of Smad7 in attenuating TGF-β-mediated fibrosis in multiple organs.

### Smad7 in immune response and inflammation

As TGF-β is a critical regulator in immune and inflammation responses [34,35], the potential function of Smad7 in immune response and inflammation has been extensively investigated. Whether Smad7 acts as an anti-inflammatory factor or as a pro-inflammatory factor varies with the context of the stimulating agent, microenvironment, tissue type, and disease model.

Smad7 has been found to possess a strong anti-inflammatory effect in various studies. Smad7 is able to suppress tumor necrosis factor α (TNFα) signaling pathway through association with TGF-β-activated kinase binding proteins (TAB2/TAB3) [36]. Consistently, transgenic expression of Smad7 in the mouse skin is able to reduce inflammatory response and nuclear factor κB (NFκB) activation [36]. In agreement with such finding, overexpression of Smad7 in the kidney could attenuate both renal fibrosis and inflammation in a mouse model of autoimmune crescentic glomerulonephritis [37]. Smad7-deficient mice developed more severe diabetic kidney injury, characterized by a significant increase in microalbuminuria, renal fibrosis and inflammation [38]. Consistently, specific deletion of Smad7 in the mouse liver enhanced inflammatory response upon alcohol administration [39].

On the other hand, Smad7 was also found to have a pro-inflammatory effect. Overexpression of Smad7 in mature T cells enhances antigen-induced airway inflammation and airway reactivity [40]. Engineered CD4+ T cells with high expression of Smad7 have accelerated T cell proliferation and activation, resulting in severe colitis in the mouse [41]. Administration of Smad7 antisense oligonucleotide leads to prevention or reversal of inflammation in a colitis mice model induced by either trinitrobenzene sulfonic acid (TNBS) or oxazolone [42], consistent with the clinical findings that the TGF-β signaling is impaired by elevated Smad7 expression in inflammatory bowel disease (IBD) in humans [43]. In addition, systemic administration of Smad7 antisense oligonucleotide was able to suppress inflammation in an experimental autoimmune encephalomyelitis (EAE) mouse model [44]. Consistently, transgenic overexpression of Smad7 in T cells could accelerate EAE and enhance T helper 1 response that was blocked by conditional deletion of Smad7 in T cells [45].

### Smad7 and tumorigenesis

TGF-β plays a dual role in cancer development, acting as a tumor suppressor in tumor initiation in an early stage but promoting tumorigenesis at a late stage by promoting epithelial-mesenchymal transition (EMT), migration, invasion and metastasis [3]. It was found that genetic polymorphisms of Smad7 are associated with human colon cancers [46,47]. In a chemical-induced mouse skin tumor model, Smad7 was found to be overexpressed [48]. These observations suggest that Smad7 might promote carcinogenesis *in vivo*. Indeed, such a notion was supported by utilizing various mice models with altered Smad7 expression levels. Overexpression of Smad7 in primary mouse keratinocytes could cooperate with oncogenic Ras to promote malignant conversion in a mouse model for squamous cell carcinoma [49]. Smad7 overexpression in the pancreas was found to induce premalignant ductal lesions [50]. Smad7 overexpression could also induce liver metastases using splenic injection of nude mice with colon adenocarcinoma (FET) cells that had overexpression of Smad7 [51]. Furthermore, overexpression of Smad7 in mouse airway epithelium under the control of a mouse Clara cell specific 10 kDa protein (CC10) promoter could promote formation of lung cancer formation upon urethane treatment [52]. Collectively, these studies indicate that Smad7 has a tumor-promoting function, most likely due to its inhibitory effect on the tumor suppressor activity of TGF-β.

Interestingly, Smad7 has also been reported to inhibit tumor formation and metastasis as revealed by various studies. Overexpression of Smad7 in breast cancer cells led to reduction in metastasis and improvement of survival in tumor-bearing mice [53]. In a human melanoma cell lines, ectopic expression of Smad7 was able to reduce tumorigenesis as analyzed by xenograft experiment in nude mice [54]. More recently, it was found that overexpression of Smad7 in T cells was able to suppress colitis-associated tumors in the mice, accompanied by elevated expression of interferon-γ (IFNγ) and accumulation of cytotoxic CD8+ and Natural Killer (NK) T cells in the tumors and peritumoral areas [55]. These studies, therefore, have pinpointed a complex role of Smad7 in tumorigenesis.

### Smad7 in epithelium

The physiological functions of Smad7 in the skin have been demonstrated by a number of studies. Keratin K5 promoter-induced overexpression of Smad7 specifically in mouse epidermis shows multiple epithelial and developmental abnormalities at birth, exhibited as corneal defects in the eyes, aberrant hair follicle morphogenesis in the skin, epidermal hyperproliferation in the digestive tract and severe thymic atrophy [16,56]. The epithelial hyperplasia arisen in these Smad7 transgenic mice might be contributed to increased cell proliferation and aberrant apoptosis. Another mechanistic possibility could be dysregulation of Wnt/β-catenin signaling, as Smad7 was revealed to antagonize Wnt/β-catenin signaling by accelerating β-catenin degradation such that skin differentiation was shifted from forming hair follicles to sebaceous glands [16]. In addition, transgenic expression of Smad7 in the mouse skin could reduce inflammatory response and NFκB activation [36]. Collectively, these studies reveal that Smad7 plays an important role in the differentiation, fibrosis and inflammatory response of the epithelium.

### Smad7 in metabolism

It was recently discovered that Smad7 plays an important role in β-cells, the insulin secreting cells in the pancreatic islets [57]. Conditional overexpression of Smad7 under the promoter of Pdx1, a gene that is specifically expressed in pancreatic progenitor cells as well as in adult β cells, could disrupt endocrine cell differentiation in embryonic stage and cause overt diabetes when Smad7 was conditionally induced in adult β-cells [57]. This study indicates that TGF-β signaling is critical for homeostasis of adult β-cell functions, as the reduction in insulin secretion and hyperglycemia caused by Smad7 overexpression could be rescued by restoration of TGF-β signaling in the β-cells [57]. In a streptozotocin-induced diabetic mouse model, deletion of Smad7 could accelerate diabetic renal injury, likely caused by increases in TGF-β signaling and NF-κB-mediated signaling pathway [38]. Consistently, overexpression of Smad7 in diabetic rats could attenuate renal fibrosis and inflammation [38]. Furthermore, hepatic deficiency of Smad7 in the mice accelerates alcohol-induced liver steatosis, likely caused by blunting of ethanol-induced production of alcohol dehydrogenase 1 (ADH1) and upregulation of critical genes involved in lipogenesis [39]. Collectively, these studies indicate that Smad7 may have a functional role in metabolic control.

## Conclusions and perspectives

In the past decades, the physiological roles of Smad7 have been elucidated by using different mouse models with either overexpression or deletion of Smad7 in different tissues/organs (Table 1). These studies have pinpointed various functions of Smad7 in a large array of biological processes including early embryonic development, fibrosis of many organs, skin cell differentiation, regulation of immune response and inflammation, tumorigenesis, and metabolic control (Table 1). A few conclusions could be drawn based on these *in vivo *studies. Firstly, it appears that a balanced expression level of Smad7 is necessary for maintaining homeostasis during embryonic development and for proper functionality of many organs. For example, dysregulated Smad7 expression may cause severe developmental defects in cardiovascular morphogenesis, lung branching, hair follicle morphogenesis, and eye development. Secondly, the major convincing evidence in terms of using overexpression of Smad7 as a potential therapy is the protective roles of Smad7 on fibrotic damage through blocking TGF-β-induced ECM production. Thirdly, the roles of Smad7 in the regulation of immune response and inflammation vary depending on the type of tissues/cells and the nature of inflammatory stimuli. Fourthly, the function of Smad7 on tumorigenesis is complex, dependent on the tumor type and model system used in the study. Whether Smad7 has a definitive role in tumor progression and metastasis awaits application of more sophisticated mouse models focused on analyzing late-stage tumor development. Finally, it is noteworthy that Smad7 may have other important physiological functions yet to be discovered, such as its roles in different facets of metabolic control. With the advances of cutting-edge technology, it is anticipated that Smad7 will continue to be an intriguing molecule that will be vigorously investigated in the future, especially considering its close relevance to human diseases.

**Table 1:** Overview of mouse studies with altered expression of Smad7

Organ (Tissue)	Modification of Smad7	Functions or phenotypes	Proposed mechanisms	Related disease(s)	Ref
Zygote	Overexpression	The embryos do not develop beyond 2-cell stage	Inhibiting TGF-β and BMP signaling	none	[14]
Lung	Overexpression	Anti-fibrosis, anti-inflammation, tumorigenic, involved in lung branching morphogenesis and epithelial differentiation	Blocking synthesis of ECM, preventing NFκB activation and inhibiting EMT	Lung fibrosis, neonatal hyperoxia, allergic asthma and lung cancer	[15,26,52]
Eyes	Over-expression	Anti-fibrosis, anti-inflammation, and improving healing from chemical burn injury	Blocking EMT and accelerating healing after injury	Retinal pigment fibrosis	[30,58,59]
Skin	Overexpression	Shifting skin differentiation from hair follicles to sebaceous glands, accelerating squamous cell carcinogenesis, tumorigenic, anti-inflammation	Suppressing Wnt/β-catenin signaling, inducing EGF-like growth factors expression, blocking NFκB signaling	Scleroderma, skin cancer, inflammation	[16,36,49,56]
Liver	Overexpression Knockout	Anti-fibrosis, anti-inflammation, inhibiting EMT and apoptosis, reducing alcoholic liver injury	Blocking ECM production, anti-oxidative stress, activating ADH1, suppressing alcoholic steatosis	Liver fibrosis, cirrhosis, alcoholic fatty liver	[32,33,39]
Pancreas	Overexpression	Abolishing β-cell differentiation in embryo, inhibiting insulin secretion in adults, anti-fibrosis, inducing early tumorigenesis	Inhibiting TGF-β signaling, pro-proliferative	Pancreatic fibrosis, pancreatitis, pancreatic cancer, diabetes	[29,50,57]
Kidney	Overexpression Knockout	Anti-fibrosis, anti-inflammation, protecting against diabetic injury	Reducing ECM accumulation, blocking NFκB signaling	Nephritis, kidney fibrosis, diabetic kidney injury	[27,28,37,38]
Colon	Overexpression Knockdown	Enhancing inflammatory reaction, increasing cancer metastasis	CD4+ T cell overactivation and resistance to regulatory T cell, reduced growth inhibition and apoptosis	IBD, colitis, colorectal cancer	[42,51]
CNS	Overexpression Knockdown	Pro-inflammatory, craniofacial and cardiac defects	Promoting T cell activation, blocking Wnt, BMP and TGF-β signaling	Autoimmune encephalomyelitis, craniofacial defects	[18,44]
Immune cells	Overexpression Knockout	Involved in proliferation of T cell and B cell response to antigen stimulation, pro-inflammatory, tumor suppression	Involved in immune cell maturation, proliferation and activation	Allergic asthma, autoimmune disorder, colitis, colon cancer	[19,40,41,45,55]
Bone	Overexpression	Inhibiting chondrocyte differentiation	Down-regulating p38 MAPK pathway	Joint disease and repair	[17]
Systemic	Knockout	Important for early heart development and B cell function	Apoptosis, inhibiting TGF-β signaling	Congenital heart defects	[19,20]

## Competing interests

None of the authors had any financial and personal relationships with other people or organisations that could inappropriately influence (bias) their work.

## Authors' contributions

LZ, SC and YC wrote the paper. All authors read and approved the final manuscript.

## Authors information

Susie Chen was a summer student from University of Washington at Seattle.
